# The hamstring stretch angle: a screening and monitoring tool for canine fibrotic myopathy

**DOI:** 10.3389/fvets.2025.1600602

**Published:** 2025-05-07

**Authors:** Kate Elizabeth Birdwhistell, Erin Miscioscia, Christina Montalbano, Jennifer Repac

**Affiliations:** Department of Comparative, Diagnostic & Population Medicine, College of Veterinary Medicine, University of Florida, Gainesville, FL, United States

**Keywords:** fibrotic myopathy, German Shepherd, hamstrings flexibility, goniometry, goniometry method

## Abstract

**Introduction:**

Fibrotic myopathy of the gracilis, semimembranosus, and semitendinosus muscles is an uncommon disease in dogs and has been primarily described in working line German Shepherds. Fibrotic myopathy can dramatically shorten the working life of military working dogs and is thus an economically important disease given the substantial cost of training. The primary objective of this study was to establish reference ranges for hamstring stretch angles from unaffected German Shepherds and unaffected retrievers (Goldens and Labradors). The secondary objective was to compare these unaffected dog hamstring stretch angles to those from German Shepherds affected with fibrotic myopathy.

**Methods:**

Thirty dogs (20 angles per group) were used to compare a total of 60 hamstring stretch angles. The hamstring stretch angle was defined as the angle of stifle extension while the hip was held in hyperflexion. Twenty unaffected German Shepherds and retrievers were prospectively recruited for inclusion in this study to establish normal reference ranges and compared to retrospective measurements of German Shepherds affected with fibrotic myopathy. Receiver operator characteristic curves were generated to establish a cut off value for fibrotic myopathy screening.

**Results:**

The mean hamstring stretch angle was 147° for the unaffected shepherds and retrievers and 109° for the affected German Shepherds. There was no significant difference in the hamstring stretch angles between unaffected German Shepherds and the retrievers. There was a mean 37° difference between the affected German Shepherd group when compared to the unaffected German Shepherds and the retrievers (*p* < 0.0001). A hamstring stretch angle of 136° was determined to be the cutoff value for further fibrotic myopathy screening with a sensitivity of 100% and specificity of 100%.

**Conclusion:**

The hamstring stretch angle may serve as a quick, inexpensive, and noninvasive method to screen for fibrotic myopathy of the gracilis, semimembranosus, or semitendinosus muscles and future research is indicated to evaluate its use as a monitoring tool for disease progression.

## Introduction

Fibrotic myopathy is a disease in which normal muscle tissue is replaced with fibrotic tissue, resulting in contracture and loss of tissue elasticity (1). Fibrotic myopathy affecting the hamstring muscles (gracilis, semimembranosus, or semitendinosus) is a rare disease in dogs and has been reported in German Shepherds, Belgian Malinois, St. Bernard, Doberman Pinscher, Old English Sheepdog, and Rottweilers (1–4). However, the majority of reported cases affect young male working line German Shepherd dogs (5, 6). The hamstrings are the primary muscle group responsible for stifle flexion and hip extension (7). This muscle group is important for propulsive movements. There are numerous posited causes of fibrotic myopathy such as: muscle trauma, fractures, compartment syndrome, neuropathy, infectious causes, immobilization, and immune mediated processes, but the etiology remains unknown (8, 9).

Fibrotic myopathy of the hamstrings can be readily diagnosed via the observed changes in gait pattern characterized by a shortened stride in the affected pelvic limb with a rapid, elastic medial rotation of the paw, external rotation of the hock, and internal rotation of the stifle during mid to late swing phase (2). Palpation of the muscle bellies themselves reveals a firm band of tissue running from the proximal inner thigh to muscle insertion at the caudomedial stifle (4, 10). Mechanical lameness is often the primary complaint and is generally not associated with pain.

Both surgical and medical management options for fibrotic myopathy have been explored. Surgical management alone has been unrewarding as dogs treated with a tenotomy or myectomy have either had persistent or recurrent lameness within a few months (2, 8). Surgical management followed by rehabilitation therapy has been reported to have a fair to guarded prognosis. Medical management options include: extracorporeal shockwave therapy, intra-lesional adipose-derived mesenchymal stem cell injections, immunosuppressive doses of corticosteroids, photobiomodulation therapy, therapeutic ultrasound, cross-fiber friction massage, passive range of motion, and controlled exercise (6, 11, 12).

Musculoskeletal ultrasound is the most commonly employed method of diagnosis and monitoring of fibrotic myopathy. However, this method is expensive, moderately invasive (often requires sedation), and requires specialized skill in musculoskeletal ultrasound. Goniometry is a relatively reliable and inexpensive way of measuring hamstring flexibility in humans, but reference ranges have not been established in the dog (13). The primary aim of this study was to establish reference ranges for hamstring stretch angles obtained from normal German Shepherds and retriever breeds. The second aim of this study was to compare the normal retriever and German Shepherd hamstring stretch angles with measurements of German Shepherds affected with fibrotic myopathy of the gracilis, semimembranosus, or semitendinosus muscles as diagnosed via musculoskeletal ultrasound. We hypothesized that fibrotic myopathy affected German Shepherds would have significantly smaller hamstring stretch angles than the unaffected German Shepherds and retrievers. If these groups are readily differentiated this measurement can serve as a fast, noninvasive, and inexpensive means of screening and monitoring the progression of fibrotic myopathy.

## Methods

### Study recruitment, inclusion, and exclusion criteria

This study was approved by the University of Florida Institutional Care and Use Committee (IACUC Approval #202400000163) and by the College of Veterinary Medicine Hospital Research Review Committee (VHRCC Approval#2024-17). Informed client consent was collected from all participants before enrollment. Control (unaffected) dogs were recruited to the ### College of Veterinary Medicine between July 15, 2024 and August 15, 2024 by soliciting staff, students, clients, and faculty of the College of Veterinary Medicine. The inclusion criteria were German Shepherds and retriever breeds (Golden or Labrador Retrievers) between one and 10 years of age. Dogs were excluded if they had signs of orthopedic or neurological disease in their hindlimbs on complete orthopedic or neurologic exam (performed by a small animal sports medicine and rehabilitation resident) or were averse to handling.

Measurements of German Shepherds affected by fibrotic myopathy were collected retrospectively by reviewing medical records of dogs that presented to the University of Florida College of Veterinary Medicine between July 26, 2021 and August 9, 2024. All dogs were diagnosed with fibrotic myopathy based on physical exam findings and confirmed by musculoskeletal ultrasound. During this time period, all clinicians used the same measuring technique as was done for the prospectively enrolled dogs.

### Hamstring stretch angle measurement

**Figure 1:**
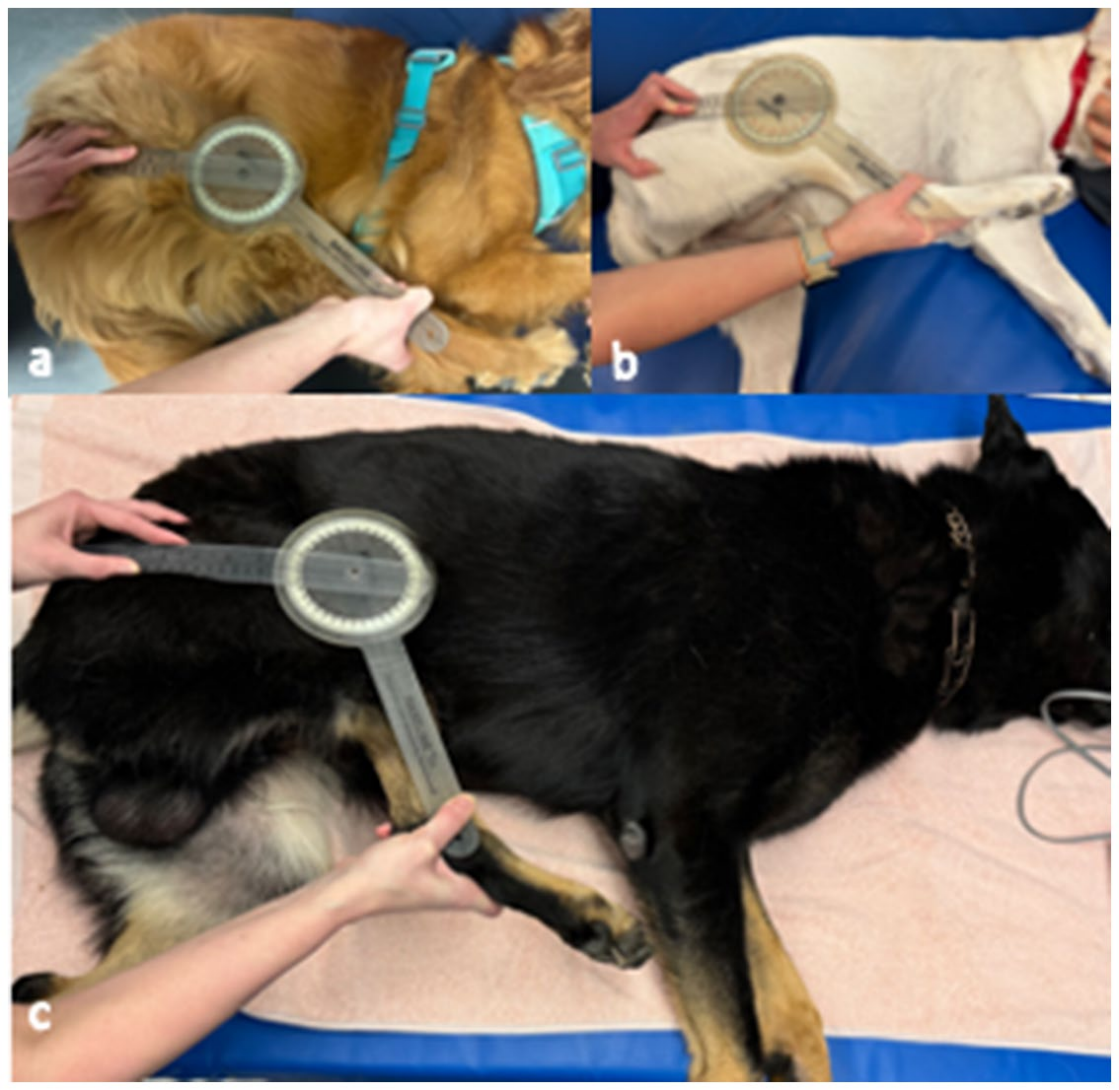
Hamstring stretch angle measurement in **(a)** normal Golden Retriever, **(b)** normal Labrador Retriever, **(c)** German Shepherd with fibrotic myopathy. The goniometer was stabilized against the greater trochanter of the femur at the hip, against the center of the stifle, and against the lateral malleolus. The stifle was extended to the greatest degree (with the hip held in hyperflexion) without allowing the pelvis to rotate.

The hamstring stretch angle was defined as the angle (in degrees) of maximum stifle extension while in hip hyperflexion (maximum hip flexion before ventroflexion of the pelvis) using bony landmarks as described previously (14, 15). Specifically, the center of the goniometer was focused on the center of the stifle, one arm held against the greater trochanter of the hip, and one arm held against the lateral malleolus. The hamstring stretch angle was measured bilaterally for all groups. All dogs were measured using a 12 inch Baseline goniometer (Fabrication Enterprises, Elmsford, NY) while lying in lateral recumbency (Figure 1). Measurements of unaffected dogs were collected without sedation at one visit by a single examiner (KB). Some of the affected German Shepherd dogs were measured on multiple occasions. For dogs that were measured on multiple occasions, the most recent measurements were selected and used for analysis. All but two of the affected German Shepherd dogs were sedated during measurements for reasons unrelated to this study.

### Statistics

A one-way analysis of variance (ANOVA) was used to assess the effect of dog type (unaffected German Shepherd, unaffected retriever, or fibrotic myopathy affected German Shepherd) on the hamstring stretch angle. Each leg was included as an independent measurement (*n* = 20 total for each group). Pairwise comparisons were made between groups using a Tukey’s multiple comparison test. The significance level (*α*) was set to 0.05. Effect size was reported as a Pearson correlation coefficient defined as small if *r* varies around ±0.2, medium if *r* varies around ±0.5, and large if *r* varies around ±0.8 as described previously (16). To establish a cut-off value for fibrotic myopathy screening, a receiver operator characteristic (ROC) curve was generated. All analyses were completed using Prism 10 software (GraphPad, La Jolla, CA).

## Results

### Study population

Thirty dogs were used in the study including: 10 unaffected German Shepherds, 10 unaffected retrievers, and 10 German Shepherds affected with fibrotic myopathy of the gracilis, semitendinosus, or semimembranosus muscles. In the retriever breed group, five were golden retrievers and five were Labrador Retrievers. Four retrievers were neutered males, five were spayed females, and one was an intact male. The mean age of the retriever group was (3.7 ± 1.7 years). Among the unaffected German Shepherds, five were intact males, four were intact females, and one was a spayed female. The mean age of the unaffected German Shepherd group was (3.3 ± 2.2 years). In the affected German Shepherd group, two were spayed females, five were intact males, and three were neutered males. The mean age of the affected German Shepherd group was (6.7 ± 3.1 years). Two of the affected dogs were protection sport dogs, none of the rest were working or sporting dogs.

### Hamstring stretch angles

**Figure 2:**
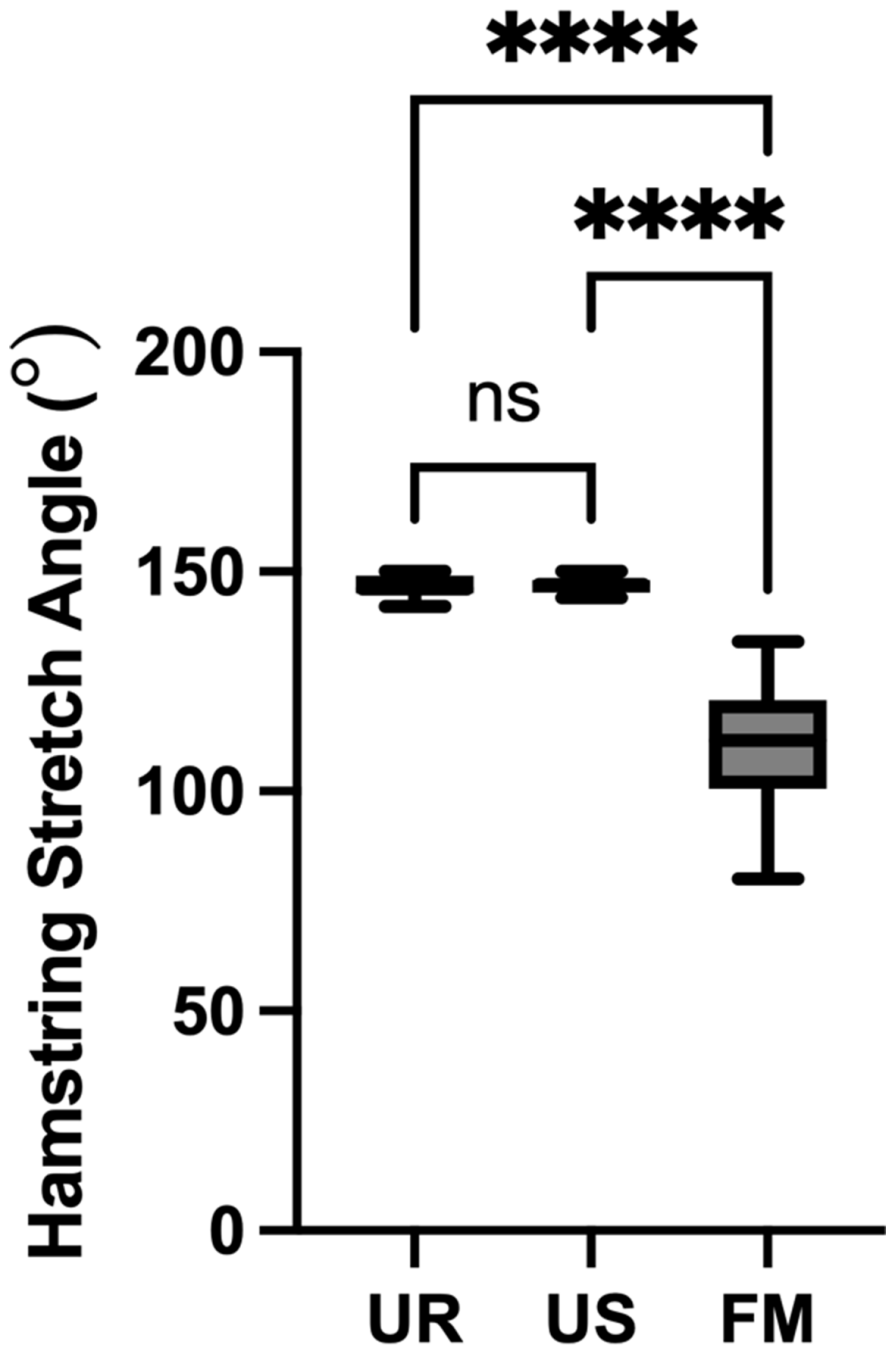
One-way ANOVA comparison of hamstring stretch angles from German Shepherds affected with fibrotic myopathy (FM), unaffected German Shepherds (US), and unaffected retrievers (UR). There was a significant difference between the fibrotic myopathy shepherds and the unaffected shepherds and retrievers (^****^p < 0.0001). However, there was not a significant difference between the unaffected shepherds and retrievers (ns, not significant).

**Figure 3:**
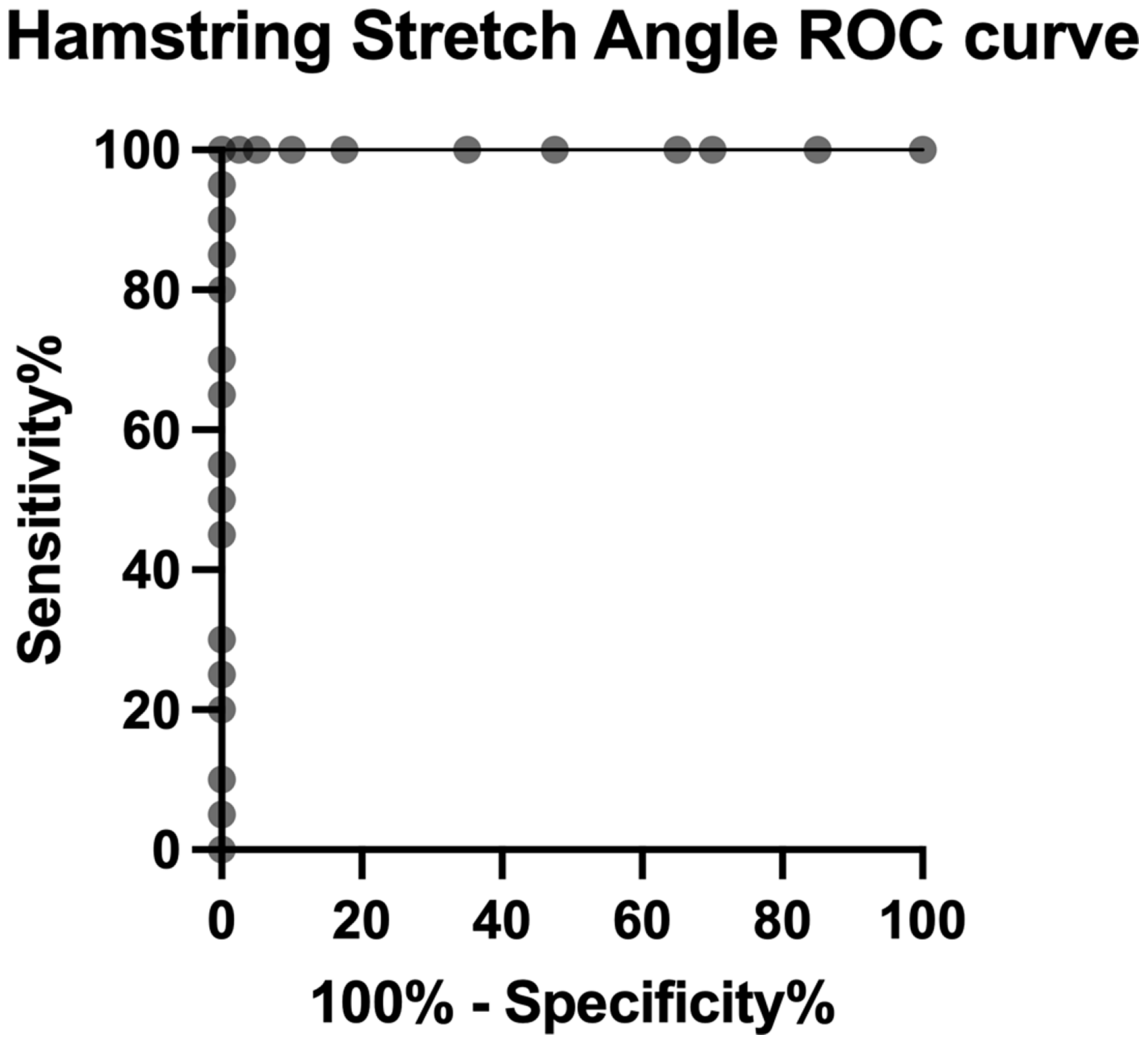
Receiver operator characteristic curve for the hamstring stretch angle test to determine fibrotic myopathy disease status.

The minimum angle, maximum angle, average angle, and standard deviation for each group is noted in Table 1. There was a significant effect of dog group (fibrotic myopathy affected German Shepherds, unaffected retrievers, or unaffected German Shepherds) on the hamstring stretch angle (*F* = 107.8, *p* < 0.0001, Figure 2). The effect size was large with an associated Pearson correlation of *r* = 0.88. There was no significant difference between unaffected German Shepherds and unaffected retrievers (*p* = 0.9981). The mean difference between the unaffected retrievers and the fibrotic myopathy affected German Shepherds was 37.1° (*p* < 0.0001). The mean difference between unaffected German Shepherds and fibrotic myopathy German Shepherds was 37.3° (*p* < 0.0001). The area under the curve of receiver operator characteristic curve was 1 meaning that the ROC curve was ideal (17). The associated cut off value for further fibrotic myopathy screening was determined to be 136° with a sensitivity of 100% and specificity of 100% (Table 2 and Figure 3).

**Table 1:** Descriptive statistics of expected hamstring stretch angles in German shepherds affected with fibrotic myopathy, unaffected German shepherds, and unaffected retrievers.

Descriptive Statistics	Unaffected shepherds (*n* = 20)	Unaffected retrievers (*n* = 20)	Fibrotic myopathy more affected limb (*n* = 10)	Fibrotic myopathy less affected limb (*n* = 10)
Minimum angle	144	138	80	100
Maximum angle	150	150	120	134
Mean angle	147	147	102	116
Standard deviation	1.8	3.3	14.4	12.4

Descriptive statistics were reported according to which limb was more clinically affected for the German Shepherds with fibrotic myopathy.

**Table 2:** Sensitivities and specificities of the hamstring stretch angle test at different hamstring stretch angles (HAS).

HAS (°)	Sensitivity (%)	Specificity (%)
82.5	5	100
87.5	10	100
95	20	100
101	25	100
102.5	30	100
106	45	100
111.5	50	100
114.5	55	100
116.5	65	100
119	70	100
120.5	80	100
123	85	100
128.5	90	100
133	95	100
136	100	100
140	100	97.5
142.5	100	95
143.5	100	90
144.5	100	82.5
145.5	100	65
146.5	100	52.5
147.5	100	35
148.5	100	30
149.5	100	15

## Discussion

Goniometry has been established as an inexpensive, readily available, and reliable method for determining the severity and progression of joint disease in dogs, humans, cows, and other species (18–21). Goniometry is also useful for the assessment of the soft tissues surrounding the measured joints (22). In this study a universal plastic goniometer was used to assess the hamstring stretch angles. There are some notable limitations of goniometry measurements. A previous study by Freund et al. (23), has shown that the universal plastic goniometer has less variability than other novel goniometers and goniometric measurement devices. Additionally, variations in the joint mechanics and the difficulty in maintaining contact with the necessary landmarks in some dog breeds may limit the utility of goniometry in veterinary medicine (24).

Previous studies have reported the normal ranges of stifle extension in several dog breeds including, Golden Retrievers, Doberman Pinschers, Belgian Malinois, Boxers, Border Collies, Rottweilers, German Shepherds, and Labrador Retrievers (15, 25, 26). However, all previous studies measured stifle extension in a standing or neutral hip angle. In contrast, this study measured stifle extension during hip hyperflexion to evaluate the elasticity of the hamstrings. Classically, the gracilis and semitendinosus complex is stretched via stifle extension, hip flexion, and hip abduction (27). The authors elected to exclude hip abduction for this study given the difficulty in standardizing hip abduction in conjunction with stifle extension and hip flexion. Even so, there were clear differences between the fibrotic myopathy group and the unaffected retriever and unaffected German Shepherd groups.

The results of this study supported our hypothesis that fibrotic myopathy affected German Shepherds have significantly smaller hamstring stretch angles when compared to unaffected shepherds and retrievers. German Shepherds with fibrotic myopathy had an average of 37° smaller hamstring stretch angle compared to both unaffected shepherds and retrievers. Previous studies have shown that a 10° loss of range of motion in the stifle joint can result in clinical lameness (28). Stifle range of motion has been shown to vary more than other joints of the pelvic limb (15). Additionally, multiple studies have demonstrated breed differences in stifle range of motion (28–32). However, the hamstring stretch angles in this study did not differ significantly among normal dogs in the breeds examined.

This study has several limitations. First, the sample size was small with only 10 dogs per group which may have an effect on the obtained receiver operator curve. Although the difference between the fibrotic myopathy affected dogs and unaffected dogs was clearly significantly different with the current sample size, no difference was found between the unaffected German Shepherds and retrievers. This lack of difference among normal dogs could be attributable to type II statistical error due to the small sample size. Nevertheless, a previous study by Sebanci et al. (26) comparing stifle extension in a neutral hip angle found a similar overlap in the angles of normal German Shepherds, Labrador Retrievers, and Golden Retrievers. Furthermore, the majority of the fibrotic myopathy dogs were sedated during measurements, unlike the normal unaffected dogs. However, the authors would anticipate that relaxation of muscles during sedation would result in overestimation of stifle extension angles (33). Additionally, there were a disproportionate number of male German Shepherds included in the fibrotic myopathy group compared to the unaffected German Shepherd and retriever groups. However, this difference is consistent with previous literature which reports a disproportionate prevalence in male working line German Shepherds (5). Finally, the measurements of the fibrotic myopathy affected German Shepherd were collected retrospectively. Therefore, not all of these measurements were collected by the same clinician. Inter- and intra-rater reliability testing has yet to be completed to know the degree of agreement between observers. However, this study was intended to serve as a first inquiry into this screening method and those validation assessments should come as a next step.

In conclusion, German Shepherd dogs with fibrotic myopathy of the gracilis, semimembranosus, or the semitendinosus muscles demonstrated significantly smaller hamstring stretch angles than unaffected shepherds and retrievers. Therefore, the hamstring stretch angle may be an effective and noninvasive tool to screen for fibrotic myopathy. However, further studies are needed to investigate the utility of this measurement for monitoring disease progression long term.

## Data Availability

The raw data supporting the conclusions of this article will be made available by the authors, without undue reservation.
